# A Curious Case of HLH: A Case Report of Hemophagocytic Lymphohistiocytosis Secondary to Atezolizumab

**DOI:** 10.1155/crh/7839972

**Published:** 2026-04-15

**Authors:** Matthew Sawyer, Nishkala Neela, Avery Pennywell, Vasanthan Kuppuswamy, Dale Okorodudu

**Affiliations:** ^1^ Department of Internal Medicine, University of Texas Southwestern Medical Center, Dallas, Texas, USA, utsouthwestern.edu; ^2^ Department of Internal Medicine, Texas Tech University Health Sciences Center El Paso, El Paso, Texas, USA, ttuhsc.edu

## Abstract

A 65‐year‐old man presented to the hospital with multiorgan failure in the setting of recently being started on atezolizumab for his small cell lung cancer. His main clinical findings were white blood cell count of 18.9 K/μL (48% neutrophils, 32% lymphocytes, 15% monocytes, and 0% eosinophils), creatinine of 10.3 mg/dL (increased from a baseline of 1.5), transaminitis with AST of 1365 and ALT of 388, total bilirubin of 2.3, a lactic acid above 11 (the upper detectable limit of our analyzer), and a serum bicarbonate of 2.8. Imaging was only notable for splenomegaly. Upon presentation, he was intubated for airway protection and admitted to the intensive care unit. Over 3 days, he was treated for severe septic shock with multiple blood pressure medications, antibiotics, continuous renal replacement therapy (CRRT), and stress dose steroids. On Day 4, his CRRT machine began clotting with a yellow, lipidic film, leading us to consider HLH. His ferritin and triglycerides were largely elevated, and hemolysis labs showed destruction of cells, making HLH the leading diagnosis. He was started on high‐dose steroids while the full interleukin panel was pending. The IL‐2 soluble receptor came back elevated, confirming the diagnosis of HLH. Before this panel returned, he received one dose of tocilizumab with high‐dose steroids before dying. This case is unique as it is the fourth documented case of secondary HLH due to the immune checkpoint inhibitor, atezolizumab. This presentation of HLH was also difficult due to the lack of fever, hepatomegaly, and cytopenias commonly seen as the presenting symptoms in HLH. Prompt initiation of treatment for HLH is critical, and due to this challenging presentation, this patient did not receive steroids and tocilizumab until Days 4 and 5, respectively.

## 1. Introduction

Hemophagocytic lymphohistiocytosis (HLH) is a syndrome that is characterized by widespread immune activation leading to severe inflammation and multiorgan failure. This inflammation causes an inappropriately excessive activation of macrophages, natural killer cells, and cytotoxic T‐cells [1]. HLH is categorized as either primary (pHLH) or secondary (sHLH), with the former being a genetic predisposition or inherited immunodeficiency and the latter being acquired due to infection, malignancy, or drug‐induced [2]. Diagnosing HLH can be difficult, as the disease often presents like septic shock. The mortality for this syndrome in the ICU is as high as 57% regardless of the etiology or treatment [3]. Treatment involves immunosuppressants such as corticosteroids and cytotoxic agents, most commonly etoposide. In this report, we describe a case of HLH secondary to the immune checkpoint inhibitor atezolizumab. This case is unique as it is only the fourth reported case of sHLH due to this immune checkpoint inhibitor [4–6]. Given the paucity of data on this adverse drug reaction, the diagnosis and treatment of HLH may have been delayed.

## 2. Case Report

### 2.1. Investigations

The patient is a 65‐year‐old male with a past medical history of Type II diabetes mellitus, hypertension, hyperlipidemia, and small cell lung cancer with metastatic disease to the brain and liver who presented to the emergency department with a chief complaint of shortness of breath. He denied cough, fever, chest pain, but did have difficulty breathing, a skin rash, and abdominal pain for 3 days. Of note, he was recently diagnosed with Stage IV metastatic small cell lung cancer, and 3 weeks prior to admission, he started treatment with cisplatin, etoposide, and atezolizumab. He received his first dose of atezolizumab three weeks prior to admission, with the second dose scheduled to be administered one week prior to admission; however, this was held due to an elevated AST of 260 and ALT of 161, for which he was prescribed prednisone 90 mg for a 7‐day course for presumed immunotherapy toxicity. He had also received a single infusion of both cisplatin and etoposide. Prior to beginning chemoimmunotherapy, he had an Eastern Cooperative Oncology Group (ECOG) performance status of 0, which means that he had normal activity levels and was independent in all daily activities.

On examination, the patient appeared cachectic and was in moderate respiratory distress while on bilevel positive airway pressure via facemask (BiPAP). His pulse was 95/minute, blood pressure (BP) was 101/51 mmHg, respiratory rate was 30 breaths/minute, and oxygen saturation was 97% while on BiPAP. He was alert and oriented, lungs were clear to auscultation, cardiovascular exam was normal, abdomen was soft and nontender with no flank tenderness. He had a pruritic papular rash around his waist.

### 2.2. Diagnosis

**FIGURE 1:**
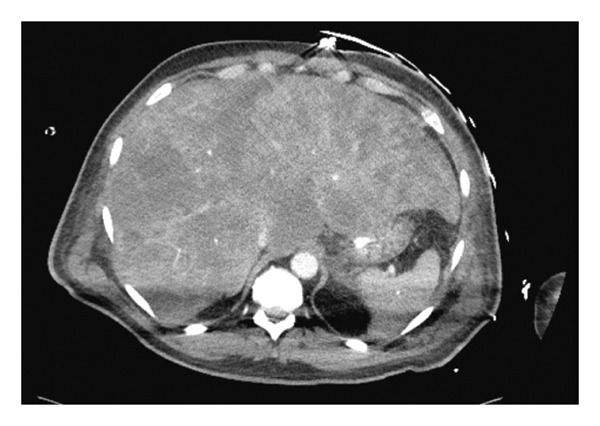
Abdominal CT scan of HLH patient with apparent hepatomegaly.

Intake labs were notable for a white blood cell count of 18.9 K/μL (48% neutrophils, 32% lymphocytes, 15% monocytes, and 0% eosinophils); creatinine of 10.3 mg/dL (increased from a baseline of 1.5 mg/dL); transaminitis with AST of 1365 U/L and ALT of 388 U/L; total bilirubin of 2.3 mg/dL; a lactic acid above 11 mmol/L, which is the upper detectable limit of our analyzer; and a serum bicarbonate of 2.8 mmol/L. An arterial blood gas revealed a pH of 6.797. The chest X‐ray was without any cardiopulmonary abnormalities, including signs of infection. We obtained aerobic, anaerobic, and fungal blood cultures as well as urine cultures, which remained negative throughout admission. The CT chest, abdomen, and pelvis did not elucidate any signs of infection and were most notable for enlargement of the liver (Figure 1). Based on the data available at that time, septic shock with multisystem organ failure (acute liver injury and renal failure) and severe immune checkpoint inhibitor toxicity were our leading differential diagnoses. Table 1 documents the trend of pertinent laboratory findings.

**TABLE 1:** Comparison of published atezolizumab‐induced HLH cases.

	Rubio‐Perez et al. [4]	Endo et al. [5]	Ota et al. [6]	
Patient demographics	67‐year‐old Caucasian male	65‐year‐old woman	80‐year‐old Japanese male	65‐year‐old male
Clinical Presentation	Active smoker, immune thrombocytopenic purpura (ITP), Stage IV lung adenocarcinoma	40 Pack‐year smoking history, metastatic lung adenocarcinoma	45 Pack‐year smoking history, Stage IIIA metastatic lung, meningitis	Metastatic Stage IV small cell lung cancer
Immunotherapy	Atezolizumab (second line)	Atezolizumab + chemotherapy	Atezolizumab (second line)	Atezolizumab + chemotherapy
Diagnostic criteria and scoring	Fever, splenomegaly, pancytopenia, hypofibrinogenemia, hemophagocytosis, bone marrow findings, hyperferritinemia, HScore: 256	Fever, splenomegaly, cytopenia, hemophagocytosis, hyperferritinemia HScore: 284	Fever, pancytopenia, hyperferritinemia, bone marrow findings HScore: NR	Pancytopenia, hyperferritinemia, hypofibrinogenemia, splenomegaly, hypertriglyceridemia, elevated soluble IL‐2r HScore: 238
Treatment	High‐dose methylprednisolone + IVIG + mycophenolate mofetil + anakinra + tocilizumab + etoposide	Prednisolone	Oral prednisolone	IV Solu‐Medrol and Tocilizumab
Outcome	Death (1 week)	Full recovery; tumor regression > 12 months	Full recovery; HLH and meningitis resolved	Death (5 days)
Unique features	First fatal atezolizumab‐only HLH case	HLH + AIHA + marker tumor regression	First HLH + immune meningitis case	HLH + concurrent sepsis

**FIGURE 2:**
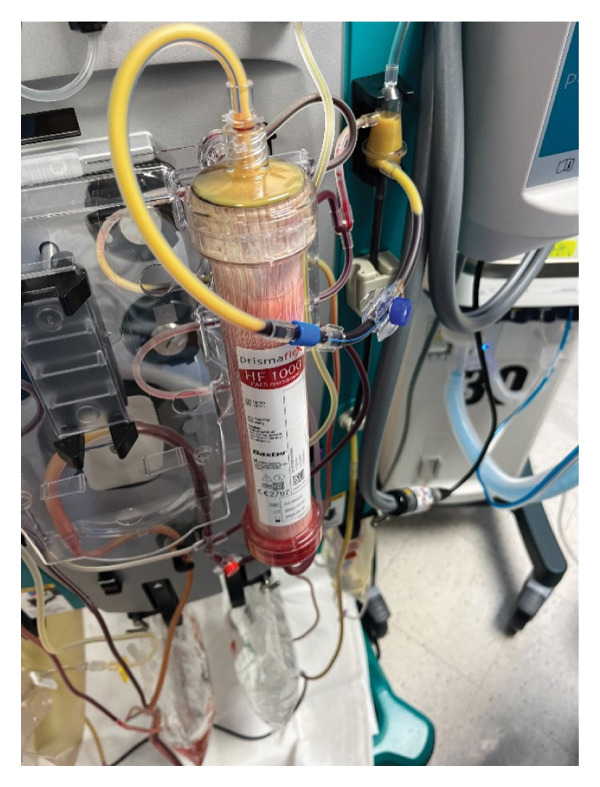
Blood of the HLH patient in a continuous renal replacement therapy filter after four days.

**FIGURE 3:**
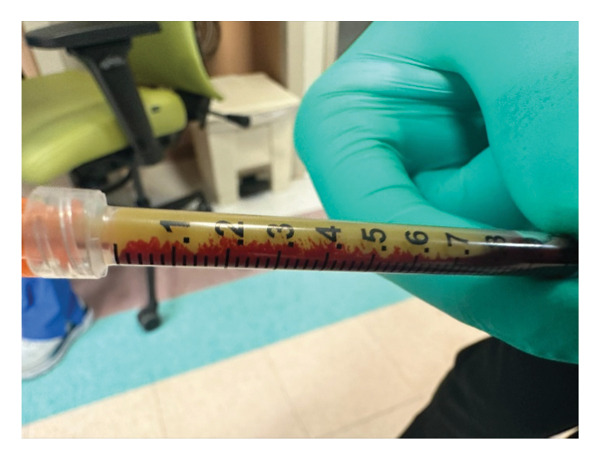
Blood of the HLH patient in a syringe.

Given our patient’s state of shock and acute renal failure, continuous renal replacement therapy (CRRT) was initiated on the day of arrival. On Day 4, the CRRT filter clotted with yellow, lipemic‐appearing blood (Figure 2). Subsequently, the patient’s nurse reported this phenomenon was also happening when his blood drawn into a syringe sat for a few minutes (Figure 3). This prompted analysis of serum triglyceride and ferritin levels, which resulted in a significant elevation at 1330 mg/dL and > 1500 ng/mL, respectively. With that, a clinical suspicion for HLH arose, and an HScore was calculated to be 238 despite scoring zero for the lack of bone marrow aspirate. This yielded an HLH diagnostic probability of 98%–99%. HLH labs were sent as seen in Table 2, and in consultation with our hematology colleagues, the diagnosis of HLH was made. The hematology team made the diagnosis of sHLH due to atezolizumab and did not ask for genetic testing of pHLH. They also deemed that a bone marrow biopsy was unnecessary as the diagnosis was clear.

**TABLE 2:** Trend of laboratory investigations.

	Day 1	Day 2	Day 3	Day 4	Day 5 (death)
Hemoglobin (g/dL)	9.1	8.0	8.8	9.4	9.4
Platelets (K/μL)	310	196	133	106	86
White blood cells (K/μL)	18.9	10.8	5.3	5.4	21.4
ALT (U/L)	388	523	> 2600	> 2600	> 2600
AST (U/L)	1365	1961	1072	1598	1737
Creatinine (mg/dL)	10.30	5.81	2.66	1.79	1.81
LDH (U/L)				1265	> 2294
Ferritin (ng/mL)				> 1500	
Triglycerides (mg/dL)				1330	
Fibrinogen (mg/dL)				127	74
D‐dimer (ng/mL)				10056	13053
Troponin (pg/mL)	28	3072	4859, 6323, 6187	7212	

This diagnosis was difficult as his presentation was consistent with septic shock, leading to poor tissue perfusion to his liver, kidneys, and heart. It was made more challenging as hematology was called in the emergency department to discuss the role of high‐dose steroids as immune checkpoint toxicity was on the differential diagnosis, but they decided that the picture was too consistent with sepsis. HLH was also lower on the differential due to the lack of fever (likely due to use of steroids and CRRT) and splenomegaly that are usually seen in HLH (Table 3).

**TABLE 3:** HLH labs.

EBV VCA early Ag IgG, nuclear Ag IgG, and VCA IgM	Positive
CMV PCR	IgG positive, IgM negative
Parvo PCR	Not detected
Interferon gamma (ref range: < 4.2 pg/mL)	6.8
Interferon 1 beta (ref range: < 6.7 pg/mL)	< 6.5
Interleukin‐10 (ref range: < 2.8 pg/mL)	403.4
Interleukin‐12 (ref range: < 1.9 pg/mL)	8.9
Interleukin‐13 (ref range: < 2.3 pg/mL)	10.1
Interleukin‐17 (ref range: < 1.4 pg/mL)	2.6
Interleukin‐2 receptor, soluble (ref range: 175.3–858.2)	9888.4
Interleukin‐2 (ref range: < 2.1 pg/mL)	2.7
Interleukin‐4 (ref range: < 2.2 pg/mL)	< 2.2
Interleukin‐5 (ref range: < 2.8 pg/mL)	< 2.1
Interleukin‐6 (ref range: < 2.0 pg/mL)	738.6
Interleukin‐8 (ref range: < 3.0 pg/mL)	183.4
Tumor necrosis factor−alpha (ref range:< 7.2 pg/mL)	17.4

### 2.3. Treatment

When first evaluated in the emergency department, he was given 1 L of lactated Ringer′s, several amps of sodium bicarbonate, started on a bicarbonate drip, initiated on broad‐spectrum antibiotics with vancomycin and cefepime, intubated, and admitted to the ICU for emergent dialysis. While in the ICU, his BP quickly became unstable, and he was started on norepinephrine, vasopressin, hydrocortisone 50 mg every 6 h, and epinephrine. Over the next few days, supportive treatment was continued with CRRT, IV vancomycin dosed per vancomycin levels, meropenem, and metronidazole 500 mg every 8 h, ventilation, and titratable norepinephrine with the occasional use of vasopressin and epinephrine to keep mean arterial pressures greater than 65 mmHg. He also had elevated troponins that were initially treated as ICI myocarditis by the cardiology team with 1 mg/kg Solu‐Medrol on Day 3.

When HLH became the leading diagnosis, he was started on 2 g IV Solu‐Medrol daily and tocilizumab 8 mg/kg every 8 hours with a plan for 4 total doses.

### 2.4. Follow‐Up and Outcomes

On Day 4, when the clinical suspicion for HLH became apparent, we sent several labs that did not come back before the patient passed away (Table 2). Before these labs came back, the decision was made to treat him with tocilizumab. He only received one dose of tocilizumab before the family made the decision to de‐escalate care and terminally extubate.

## 3. Discussion

This case of sHLH proved to be diagnostically challenging. This side effect is extremely rare, with only 3 other case reports of atezolizumab in the literature. This uncommon disease process was made more difficult due to the lack of common findings present in HLH. Even though the diagnostic criteria have changed greatly since the first guideline was published in 1991, the most common findings continue to include fever, splenomegaly, and cytopenias [7]. The H‐score is the most validated tool to aid in the diagnosis and involves adding selected points for these variables: known immunosuppression, temperature, organomegaly, number of cytopenias, ferritin, triglycerides, fibrinogen, AST level, and bone marrow aspirate. The score ranges from 0 to 337, with the best cutoff of the score being 169, corresponding to a sensitivity 93%, specificity of 86%, and accurate classification in 90% of patients [8]. This H‐score has been retroactively compared to the 2004 HLH guidelines and seems to be more powerful when diagnosing children and adults at presentation of their disease [9]. We did not have all the HLH scoring values at the time of the diagnosis, which may make the scoring system less reliable, however a score of 238, irrespective of when calculated, has a 98%–99% probability of hemophagocytic syndrome. The use of CRRT also made the diagnosis difficult as the disease progressed. CRRT is known to mask increases in body temperature during infections, and we hypothesize that CRRT mixed with stress dose steroids likely decreased our patient’s ability to spike a fever [10]. Also, the severity of disease at presentation in an immunocompromised patient led us to triage and treat the patient as severe sepsis with multiorgan failure. It was not until the patient continued to show signs of poor tissue perfusion despite adequate antibiotic coverage and negative cultures that we began to consider sHLH. Once HLH labs were sent, the diagnosis was made as the patient fulfilled five out of eight criteria: pancytopenia, hyperferritinemia, hypofibrinogenemia, hypertriglyceridemia, elevated soluble IL‐2r and the extremely elevated H‐score.

### 3.1. sHLH

As mentioned in the introduction, HLH is characterized by an uncontrolled hyperinflammatory response leading to the activation of macrophages and cytotoxic T‐cells. Atezolizumab is a Programmed death ligand 1 (PD‐L1) antibody. The Programmed cell death 1 (PD‐1) receptor is a transmembrane protein found on activated macrophages, T‐cells, and B‐cells that when bound with PD‐L1 causes downregulation of the immune system manifested by decreased Interleukin‐2 and interferon gamma production. As a PD‐L1 antibody, atezolizumab blocks the interaction between PD‐L1 and PD‐1 and inhibits this downregulation of the immune system, allowing the immune system to be activated to fight cancer cells [11]. This mechanism leads to the activation of T‐cells, B‐cells, and macrophages, which release large amounts of cytokines, further activating the immune system and potentially leading to HLH [12]. This is the proposed mechanism for immune checkpoint inhibitors causing immune‐associated adverse events of colitis, hypophysitis, hypothyroidism, skin hypersensitivity reactions, and pneumonitis.

After the diagnosis was made, the best course of treatment was another challenge. With HLH being a state of immune system overactivation, the typical treatment includes immunosuppressants in the form of high‐dose steroids and the chemotherapeutic agent etoposide. It is important to note that these treatments are standard for the pediatric population, but there is no established treatment for adults with this disease [13]. The treatment of immune checkpoint inhibitor−related HLH (irHLH) is even less studied. The treatment guidelines come from the Histiocyte Society and recommend corticosteroids plus tocilizumab and adding etoposide at Hour 48 if no clinical response occurs [14]. Tocilizumab, an IL‐6 inhibitor, is the accepted treatment, as IL‐6 is an upstream cytokine active in the early stages of HLH and required for cytotoxic T‐cell activation and survival [15]. It also has a favorable side effect profile and can be used in patients with severe renal impairment, which was needed in our case. Another prospective treatment includes anakinra [16]. Nevertheless, more reported cases are needed to better elucidate the best treatment for sHLH due to atezolizumab.

An important takeaway from this case is to keep the differential broad when triaging and managing patients receiving immune checkpoint inhibitors. Although the incidence of sHLH due to atezolizumab remains small, adverse reactions from this class of medications are unpredictable and are becoming more reported. There have been three reported cases of HLH after treatment with atezolizumab. The first case is of a 67‐year‐old man diagnosed with Stage IV lung adenocarcinoma and paraneoplastic immune thrombocytopenic purpura, who presented with HLH after treatment with his first infusion of atezolizumab. He presented with fever, pancytopenia, dyspnea, and was quickly admitted to the intensive care unit, where he passed away within a week [4]. The authors of this case believe that there may have been a correlation with the autoantibodies associated with paraneoplastic ITP for increasing the risk of other hematological adverse outcomes. The second case was of an 80‐year‐old man with Stage III lung adenocarcinoma who developed sHLH and meningitis 10 days after his first infusion with atezolizumab. His disease was caught early, and he survived with the use of corticosteroids. He was not admitted to the ICU [6]. The last known case of sHLH from atezolizumab was of a 60‐year‐old man with Stage IV lung adenocarcinoma who presented with secondary autoimmune hemolytic anemia and sHLH simultaneously. This patient was being treated with platinum doublet therapy and atezolizumab. The reactions occurred with the first infusion of atezolizumab [5]. All three of these cases were treated with high‐dose corticosteroids, and one utilized tocilizumab. In this case presentation, the diagnosis was delayed due to overlapping symptoms of shock and multiorgan failure seen in many critical care patients. This led to a delay in treatment. Our hope is that reporting this case will allow future providers to have sHLH in the differential for patients receiving atezolizumab presenting with multiorgan failure. Early recognition of this rare phenomenon will enable appropriate treatment to be started promptly.

## Funding

No funding was received for this study.

## Consent

No written consent has been obtained from the patients as there are no patient identifiable data included in this case report/series.

## Conflicts of Interest

The authors declare no conflicts of interest.

## Data Availability

Data sharing is not applicable to this article as no datasets were generated or analyzed during the current study.
